# The interplay between FOXO3 and FOXM1 influences sensitivity to AKT inhibition in PIK3CA and PIK3CA/PTEN altered estrogen receptor positive breast cancer

**DOI:** 10.1038/s41523-025-00752-9

**Published:** 2025-04-22

**Authors:** Valentina Cutano, Ming Li Chia, Eleanor M. Wigmore, Lorna Hopcroft, Stuart C. Williamson, Amanda L. Christie, Brandon Willis, James Kerr, Jenny Ashforth, Rhys Fox, Sophie D’Arcy, Lauren Bradshaw, Catherine Blaker, Cath Eberlein, Lambert Montava-Garriga, Elza C. de Bruin, Susan E. Critchlow, Kevin M. Brindle, Simon T. Barry, Susana Ros

**Affiliations:** 1 Bioscience, Early Oncology, AstraZeneca, Cambridge, UK; 2 Cancer Research UK Cambridge Institute, Cambridge, UK; 3 Early Data Science, Oncology Data Science, AstraZeneca, Cambridge, UK; 4 Bioscience, Early Oncology, AstraZeneca, Boston, MA USA; 5 Discovery Sciences, AstraZeneca, Cambridge, UK; 6 Translational Medicine, AstraZeneca, Cambridge, UK

**Keywords:** Breast cancer, Targeted therapies

## Abstract

Loss of PTEN expression, via homozygous or hemizygous deletion, is common in *PIK3CA* mutant ER + BC tumors. We assessed reduction of PTEN protein expression on AKT inhibitor capivasertib efficacy in *PIK3CA* altered tumors. In *PIK3CA* altered, PTEN protein high models, PI3Kα and AKT inhibition was effective, however ablation and partial PTEN expression reduction attenuated PI3Kαi but not AKTi efficacy, alone or combined with fulvestrant. Efficacy was FOXO3 dependent and associated with FOXM1 downregulation. FOXO3A deletion reduced response to capivasertib, and increased FOXM1 expression. Long term capivasertib exposure of ER+ BC cells upregulated FOXM1 expression. Downregulating FOXM1 expression reversed resistance to capivasertib, while FOXM1 overexpression reduced capivasertib efficacy. Collectively this suggests the AKT-FOXO3-FOXM1 axis plays a pivotal role in response to AKTi in ER+ breast cancer with *PIK3CA* mutations with and without expression of PTEN, that FOXO3 expression loss can mediate resistance, and that FOXM1 downregulation is a potential biomarker of response.

## Introduction

The PI3K-AKT signalling pathway plays a fundamental role in cell growth, proliferation, metabolism, and survival^1^. Gain of function mutations in *PIK3CA*, the gene encoding phosphoinositide-(3)-kinase α (PI3Kα), occurs in ~30–40% of advanced estrogen receptor positive (ER+) breast cancers and has been associated with poor prognosis^2^. Loss of function mutations in the tumour suppressor *PTEN*, or homozygous deletion of *PTEN* are observed in 5–10% of ER+ breast cancers and genetic activation of AKT-1 occurs in 5–7% of ER+ breast cancers^3–5^. These genetic alterations also contribute to the broader activation of PI3K-AKT signalling in ER+ BC^6^.

Targeting PI3K-AKT signalling in ER+ breast cancer is an important therapeutic strategy. Inhibiting PI3Kα signalling with the PI3Kα inhibitor (PI3Kαi) alpelisib in combination with the ER degrader fulvestrant improved progression-free survival in patients with confirmed tumour-tissue *PIK3CA* mutations^7^. More recently the AKT inhibitor (AKTi) capivasertib combined with fulvestrant also increased benefit versus fulvestrant alone in a broader segment of patients with tumour alterations in *PIK3CA*, *PTEN* or *AKT-1*^8^.

While PI3K-AKT signalling plays a critical role in breast cancer, a complex network of genetic alterations, and secondary or complimentary signalling and regulatory mechanisms that control pathway activity also influence benefit to different PI3K therapeutic approaches targeting this pathway^9,10^. In addition to activation through genetic alterations in pivotal genes, PI3K-AKT signalling is activated by growth factors such as heregulin, EGF, IGF, and bioactive lipids, or by changes in levels of regulatory proteins such as the lipid and protein phosphatase PTEN^11^. Hence there are multiple context-dependent activation and resistance mechanisms that influence response following inhibition of different enzymes on the PI3K-AKT pathway. One of the pivotal proteins is PTEN. Loss of PTEN protein through gene disruption, deletion, or protein downregulation results in activation of alternate PI3K isoforms p110β signalling (PI3Kβ)^12,13^. Loss of *PTEN* can limit the activity of drugs that target p110α particularly in *PIK3CA* altered tumours, and treatment with PI3Kβ inhibitors overcomes PTEN protein loss mediated resistance^12^.

PI3K-AKT signalling feeds into the transcription factor forkhead box O3 (FOXO3) which translocates to the nucleus when PI3K-AKT signalling is inhibited^14,15^. FOXO3 has been implicated in both sensitivity and resistance to PI3K inhibition. Extended translocation of FOXO3 to the nucleus upregulates IGF-IRS1 signalling as a positive physiological feedback loop to restore pathway activation. FOXO3 also regulates a second transcription factor FOXM1 (forkhead box protein M1)^16^. Moreover, *PIK3CA* altered tumours that have been rendered resistant to PI3Kα inhibition through loss of *PTEN* show lack of FOXM1 downregulation, suggesting FOXM1 may play a role in limiting the efficacy of agents targeting the PI3K-AKT pathway. The role of the FOXO3-FOXM1 interplay in modulating sensitivity following inhibition of AKT has been less extensively studied. Interestingly expression of the transcription factor FOXM1 is inversely correlated with PTEN expression^14,16^. Here we show that the FOXO3-FOXM1 transcription factors are pivotal drivers of response to drugs that target p110α or AKT in ER+ breast cancer models with *PIK3CA* activating mutations or in drugs that target AKT in models with *PIK3CA* activating mutation and compromised PTEN expression. Collectively these findings also emphasise the importance of FOXO3-FOXM1 as both effectors and potential biomarkers of sensitivity and resistance to PI3K-AKT pathway inhibition in ER+ BC.

## Results

### Co-occurrent *PIK3CA* and *PTEN* genetic alterations are common in ER+ breast cancer tumour biopsies

**Fig. 1:**
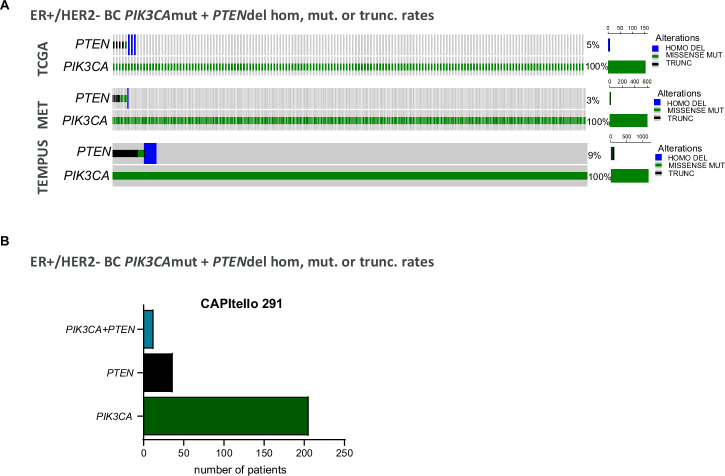
Landscape of co-alterations in *PIK3CA* and *PTEN* in ER+ /HER2- biopsies from TCGA, Metabric, Tempus and CAPItello291. **A** Using METABRIC (Molecular Taxonomy of Breast Cancer International Consortium, 1382 samples for 1382 patients), TCGA (The Cancer Genome Atlas, *n* = 459 samples for 437 patients) and TEMPUS (*n* = 3,327 samples for 3053 patients) datasets the number of ER+/HER2- breast cancer patients that present *PIK3CA* mutations and mutations or deletions in *PTEN* were calculated. **B** Results from the recent Phase 3 CAPItello 291 show that ~6% of the ER+/HER2- breast cancer *PIK3CA* biopsies present co-occurrent alterations in *PTEN* at genomic level, assessing the efficacy of the AKT-inhibitor capivasertib as an addition to fulvestrant therapy in patients with ER+ advanced breast cancer^8^.

Combining fulvestrant (an Estrogen Receptor (ER) degrader) with the PI3K-AKT signalling inhibitors alpelisib (PI3Kαi) or capivasertib (AKTi) provides benefit in ER+ breast cancer (BC) patients with *PIK3CA* altered and *PIK3CA*, *PTEN* and *AKT-1* altered tumours respectively^7,8^. Loss of, or reduction in, PTEN protein expression is common across many tumour types. Reducing PTEN protein results in activation of PI3K signalling and specifically in an increase in PI3Kβ (p110β) activity^17^, attenuating the inhibitory capacity of agents targeting PI3Kα (p110α) activity^18^. To examine in more depth the prevalence of co-occurring alterations in *PIK3CA* and *PTEN* in ER+ BC three different datasets were analysed: METABRIC (Molecular Taxonomy of Breast Cancer International Consortium)^19,20^, TCGA (The Cancer Genome Atlas)^21^ and TEMPUS (https://www.tempus.com/) (Fig. 1A). This revealed that 3 to 9% of ER+ breast cancer biopsies with mutations in *PIK3CA* had co-occurrent alterations in *PTEN* at the genomic level, with mutations, truncations or homozygous deletions in *PTEN* (Fig. 1A). To confirm these data the recent Phase III CAPItello 291 clinical trial (NCT04305496) which assessed the efficacy of the AKT-inhibitor capivasertib combined fulvestrant in patients with advanced ER+ breast cancer was also analysed^8^. This showed that ~6% of the tumours with mutations in *PIK3CA* also had mutations, truncations, or homozygous deletions in *PTEN* (Fig. 1B).

**Fig. 2:**
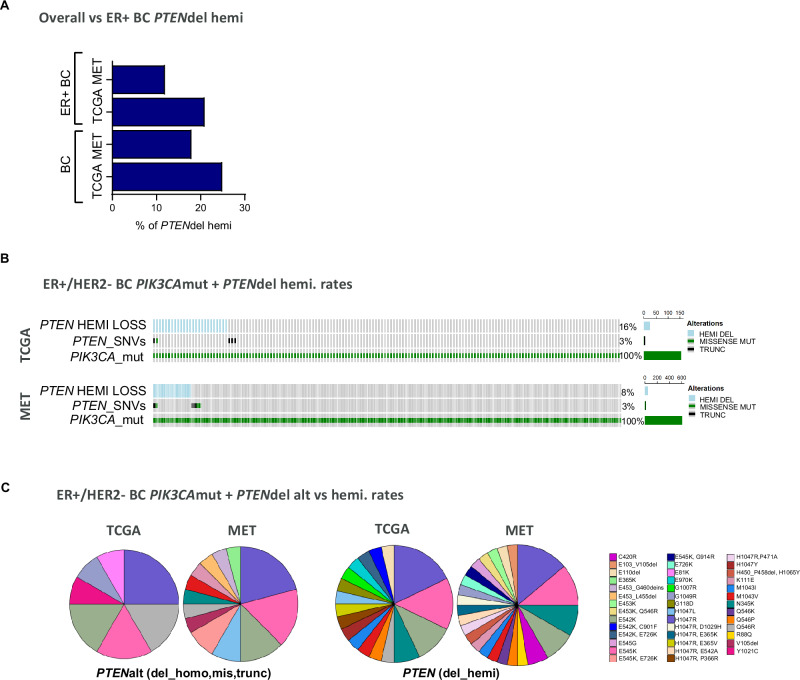
Frequency of hemizygous *PTEN* alterations in ER+ BC using METABRIC and TCGA datasets. **A** Prevalence of *PTEN* heterozygous loss in TCGA and Metabric both in the full breast cancer cohort and co-occurring within the ER+/HER2- breast cancer as above are shown as bar graphs. **B** Oncoprints for co-occurring *PTEN* heterozygous loss in *PIK3CA* mutated biopsies are shown in ER+/HER2- in TCGA and Metabric cohorts (**C**) Pie charts show the proportion of different *PIK3CA* mutations in the *PTEN*alt (homozygous loss, missense mutations and truncations) ER+/HER2- breast tumours in Metabric (*n* = 14) and TCGA (*n* = 33) and *PTEN*hemidel (hemizygous loss) in Metabric *n* = 93 and TCGA *n* = 47. Plots were generated in R version 4.3.2 with the package ‘plotly’.

*PTEN* loss of function may occur by deletion, either affecting both copies (homozygous loss) with expression absent, or one copy loss (hemizygous loss) with intermediate expression levels between homozygous loss and *PTEN* intact tumours^22^. The frequency of hemizygous loss in METABRIC and TCGA datasets was analysed (Fig. 2A). This showed that frequency of single-copy inactivation of *PTEN* in breast cancer is 18–25%, consistent with previous analysis of TCGA^22^. Moreover, *PTEN*- single copy deletion was also frequent in samples with an ER+ molecular classification (Fig. 2A). In particular, single-copy inactivation of *PTEN* was present in 8–16% of ER+/HER2- biopsies harbouring a *PIK3CA* alteration (Fig. 2B). Further analysis of the prevalence of *PTEN*alt (homozygous loss, missense mutations, or truncations) and *PTEN* hemizygous loss in ER+/HER2- BC biopsies harbouring different *PIK3CA* activating mutations present in different exons showed that co-occurrent *PTEN* alterations like mutations, truncations, homo- or hemizygous deletions are common (Fig. 2C and Supplementary Table 1). Collectively the analysis shows that alterations in *PTEN* that could drive PTEN protein loss or deficiency are common in ER+ BC biopsies with *PIK3CA* mutations, with hemizygous deletion frequency of ~18–25% and homozygous deletions being less common at frequency of ~3–9%.

### AKTi has anti-tumour efficacy in ER+ BC patient derived xenografts with *PIK3CA* alterations regardless of PTEN protein expression correlating with decreased FOXM1 expression

**Fig. 3:**
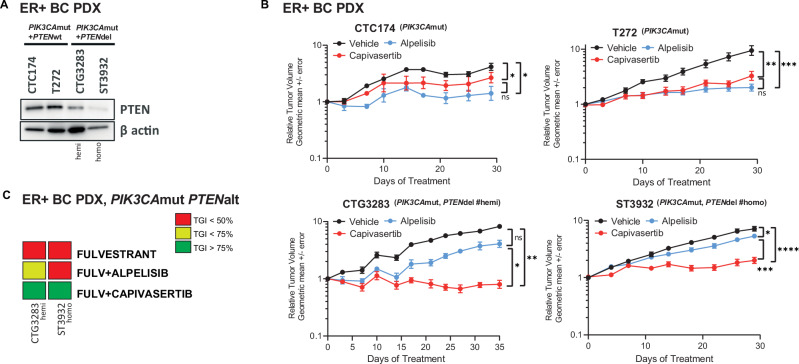
Capivasertib shows anti-tumour efficacy in ER+ BC patient derived xenografts with *PIK3CA*mut and *PTEN* homozygous or hemizygous deletions. **A** Western blot analysis of PTEN protein expression in ER+ breast cancer PDX models (CTC174, T727, CTG3283 and ST3932). βactin was used as loading control. **B** In vivo activity of capivasertib and alpelisib in ER+ breast cancer PDXs: T272 (*n* = 9), CTC174 (*n* = 4), CTG3283 (*n* = 7) and ST3932 (*n* = 10). Tumours were treated with vehicle (closed circles), 100 mg/kg capivasertib BID 4 days on 3 days off (red circles) or 25 mg/kg alpelisib QD (blue circles). Geomean tumour volumes ± SEM *p < = 0.05, **p < = 0.01, ***p < = 0.001, ****p ≤ 0.0001 are shown. **C** Tumour growth inhibition heatmap representing in vivo activity of fulvestrant or combinations with alpelisib or capivasertib in *PIK3CA*mut *PTEN* altered PDXs (CTG3283 *n* = 7 and ST3932, *n* = 8–10). Treatments with 100 mg/kg capivasertib BID 4 days on 3 days off, 25 mg/kg alpelisib QD, 5 mg/animal fulvestrant QW.

To investigate how *PTEN* loss or deficiency are common in ER+ BC biopsies harbouring *PIK3CA* alterations impacted sensitivity to PI3K-AKT pathway inhibitors we identified two ER+ breast tumour PDX models with activating mutations in *PI3KCA* and alterations in *PTEN*. The first with a homozygous deletion of *PTEN* (ST3932) resulting in reduced protein expression, and the second with a hemizygous deletion of *PTEN* (CTG3283) resulting in intermediate PTEN protein expression compared to models with no alterations in *PTEN* (labelled *PTEN*wt) (Fig. 3A)^23–27^. As anticipated, ER+ BC tumour PDX models CTC174 and T272 with activating mutations in *PI3KCA* alone were responsive to both inhibition of AKT (capivasertib) and PI3Kα (alpelisib) (Fig. 3B). In contrast in two models, CTG3283 and ST3932, with activating mutations in *PI3KCA* but reduced PTEN protein levels monotherapy treatment with an AKTi reduced tumour growth while activity of the PI3Kαi (alpelisib) was less pronounced (Fig. 3B). Both CTG3238 and ST3932 have modifications in *PTEN* with hemi and homozygous *PTEN* deletions respectively associated with the reductions in PTEN protein. Both *PTEN* altered models had little monotherapy sensitivity to fulvestrant but combining PI3Kαi with ER inhibition had modest anti-tumour effects that was attenuated relative to the combination with AKTi (Fig. 3C and Supplementary Fig. 1A). These data are consistent with homozygous and hemizygous alterations that ablate or reduce PTEN protein resulting in a reduced response to PI3Kαi but not to AKTi alone and in combination with fulvestrant, and inhibition of AKT being effective in tumours harbouring *PIK3CA* mutations regardless of PTEN protein expression (Fig. 3C and Supplementary Fig. 1A). Moreover, it suggests that reducing (rather than ablating) expression of PTEN may affect the response of *PIK3CA* altered ER+ BC PDX tumour models to PI3Kα signalling inhibition.

### FOXM1 modulation is associated with PI3K-AKT pathway inhibition in *PIK3CA* mutant ER+ BC isogenic cell lines with compromised PTEN expression

**Fig. 4:**
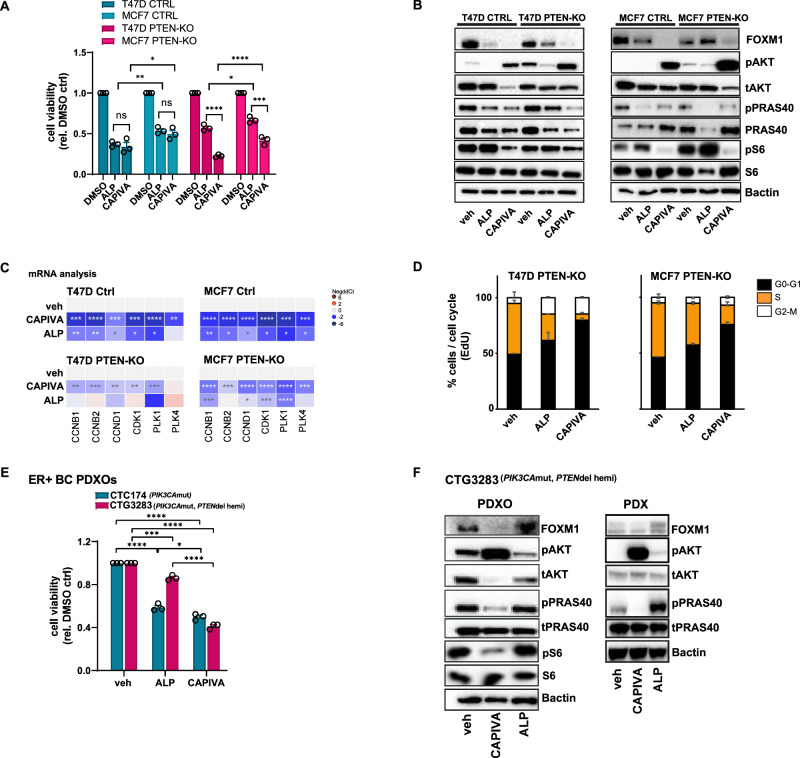
Capivasertib treatment impacts cell cycle progression of PI3Kα inhibitor acquired resistant models via FOXM1. **A** Cell viability assay of T47D CTRL, MCF7 CTRL, T47D PTEN-KO and MCF7 PTEN-KO treated for 120 h with DMSO, 0.5 μM capivasertib and 0.5 μM alpelisib and MCF7 CTRL and MCF7 PTEN-KO treated for 120 h with DMSO, 1 μM capivasertib and 1 μM alpelisib. Data were normalised to DMSO; plotted as mean ± SEM (*n* = 3). Statistical analysis 2-way ANOVA test vs vehicle-treated, alpelisib treated or capivasertib treated *p < = 0.05, **p < = 0.01, ***p < = 0.001, ****p ≤ 0.0001. **B** Western blot using MCF7 CTRL, MCF7 PTEN*-*KO, T47D CTRL and T47D PTEN*-*KO protein lysates after 96 h treatment, characterising modulation of biomarkers of the PI3K-AKT pathway. All MCF7 cells were treated with 1 μM capivasertib and 1 μM alpelisib. All T47D cells were treated with 0.5 μM capivasertib and 0.5 μM alpelisib. βactin was used as loading control. **C** Gene expression analysis relative to vehicle treatment in T47D CTRL, T47D PTEN-KO, MCF7 CTRL and MCF7 PTEN-KO of FOXM1 target genes following 5 days treatment with capivasertib and alpelisib. All MCF7 cells were treated with 1 μM capivasertib and 1 μM alpelisib. All T47D cells were treated with 0.5 μM capivasertib and 0.5 μM alpelisib. Statistical analysis 2-sided students *t*-test vs vehicle-treated *p ≤ 0.05, **p < = 0.01, ***p ≤ 0.001, ****p ≤ 0.0001 (*n* = 6). **D** Comparison of the DNA synthesis (S-phase) in MCF7 PTEN-KO and T47D PTEN-KO after 5 days treatment with capivasertib and alpelisib. MCF7 cells treated with 1 μM capivasertib and 1 μM alpelisib. T47D cells treated with 0.5 μM capivasertib and 0.5 μM alpelisib. Data are mean of 2 independent experiments ± SD. **E** Cell viability assay of PDXO models (CTC174 and CTG3283) treated with 1 μM capivasertib and 1 μM alpelisib for 5 days. Data was normalised to DMSO and plotted as mean ± SEM (*n* = 3). Statistical analysis one-way ANOVA test vs DMSO-treated *p ≤ 0.05, **p ≤ 0.01, ***p ≤ 0.001, ****p ≤ 0.0001. **F** PDXO CTG3283 characterisation and comparison with PDX CTG3283 by western blot after 5 days treatment to validate biomarker modulation upon treatment with 1 μM capivasertib and 1 μM alpelisib. βactin was used as loading control.

To explore the consequence of PTEN protein reduction in a *PIK3CA* mutant background, as well as the influence on signalling through PI3Kα and AKT more broadly, isogenic pairs of *PIK3CA* altered ER+ BC cells with and without *PTEN* deletion were used. *PTEN* was ablated using CRISPR-Cas9 technology (PTEN_KO) in the ER+ *PIK3CA*mut breast cancer cell lines T47D (H1047R) and MCF7 (E545K) overexpressing Cas9 (Supplementary Fig. 2A). Loss of PTEN protein (in polyclonal populations to maintain heterogeneity) attenuated sensitivity to the PI3Kαi alpelisib (Supplementary Fig. 2B) consistent with earlier studies^18^. Intrinsic sensitivity of *PI3KCA* and *PTEN* depleted cells to AKT inhibition reflected the intrinsic sensitivity of the single *PIK3CA* mutant cells. Both parental and PTEN protein depleted T47D cells were most sensitive to capivasertib treatment following treatment for 5 days (Fig. 4A), while growth of the MCF7 parental cells (which are intrinsically less sensitive to capivasertib) and PTEN depleted cells showed similar sensitivity to capivasertib treatment (Fig. 4A). This aligned with the effectiveness of capivasertib in reducing the phosphorylation levels of AKT pathway biomarkers, pPRAS40 and pS6, in both parental cells and PTEN-depleted cells (Fig. 4B). While total PRAS40 levels varied across treatments in both cell lines, total S6 levels were similar across the conditions (Fig. 4B). Notably while alteration of *PIK3CA* and *PTEN* did increase basal pAKT levels, it did not render cells more sensitive to AKTi than parental cells (Fig. 4A, B). Modulation of FOXM1 can be both a biomarker of response and a driver of resistance to PI3Kα inhibition in ER+ breast cancer^14^, but its role in modulating sensitivity following inhibition of AKT has been less extensively studied. Monotherapy treatment with capivasertib for 96 h decreased FOXM1 protein expression in both PTEN proficient and PTEN-KO T47D and MCF7 cells (Fig. 4B). In contrast, sustained FOXM1 expression following alpelisib treatment for 96 h was detected in *PIK3CA*mut and PTEN-KO T47D and MCF7 cells (Fig. 4B). The effect of the combination of capivasertib and fulvestrant was also assessed, which resulted in further suppression of cell growth. Response to the combination was similar in T47D and MCF7 parental and PTEN depleted cells (Supplementary Fig. 2C), although the combination treatment resulted in greater decreases in FOXM1 protein expression relative to monotherapy treatment (Supplementary Fig. 2D).

mRNA analysis of the parental T47D and MCF7 lines following capivasertib or alpelisib treatment showed downregulation of FOXM1 targets, including genes in the G2M checkpoint and E2F target pathways (Fig. 4C)^28^. In the PTEN protein depleted MCF7 and T47D lines (PTEN-KO) FOXM1 target genes were significantly downregulated following capivasertib versus vehicle or alpelisib treatments (Fig. 4C). Moreover, impact on cell cycle progression was assessed by EdU incorporation. Fewer cells in S-phase and slightly more cells in G0-G1 or G2-M phase were detected in capivasertib treated compared to vehicle or alpelisib treated PTEN protein depleted cells (Fig. 4D).

To determine whether the impact of reduction in PTEN protein on treatment response also occurred when PTEN was reduced rather than ablated, patient derived xenograft organoids (PDXOs) were generated by dissociating fragments of the CTC174 PDX with *PIK3CA*mut and *PTEN*wt and the CTG3283 PDX with *PIK3CA*mut and hemizygous *PTEN* loss (Supplementary Fig. 3A and Supplementary Fig. 3B). The status of ER in the PDXO models was maintained when compared to the parental PDX tumour models (Supplementary Fig. 3C). After 5 days of monotherapy treatment, both PI3Kα (alpelisib) or AKT (capivasertib) inhibition decreased *PIK3CA*mut CTC174 PDXO cell viability relative to DMSO control (Fig. 4E). In the *PIK3CA*mut, low PTEN expression (hemizygous deletion) CTG3283 derived PDXO model AKTi significantly decreased cell proliferation compared to PI3Kαi treatment consistent with the in vivo data (Fig. 3B). Effects were similar to those observed in PTEN-KO MCF7 and T47D lines (Fig. 4A). Consistent with the lack of effect on proliferation, FOXM1 expression was sustained following treatment with a PI3Kαi in both the PDXO derived from CTG3283, and the corresponding PDX samples (Fig. 4F). In contrast, FOXM1 was decreased in both the CTG3283 PDXO and PDX models following treatment with an AKTi. This was consistent with decreased phosphorylation of AKT pathway biomarkers e.g. pPRAS40 and pS6 in the PDXOs and pPRAS40 in the PDX models (Fig. 4F). Results parallel to those observed for the PTEN-KO MCF7 and T47D lines (Fig. 4B). These data suggest that direct modulation of PTEN protein expression in *PIK3CA* mutant ER+ BC models can modify the response to agents targeting the PI3K-AKT pathway, and that FOXM1 modulation can be a mechanistic biomarker informing on effective pathway inhibition.

### Lack of AKT mediated regulation of FOXO3 and sustained FOXM1 expression drives capivasertib resistance in *PIK3CA*-mutant *PTEN*-KO models

**Fig. 5:**
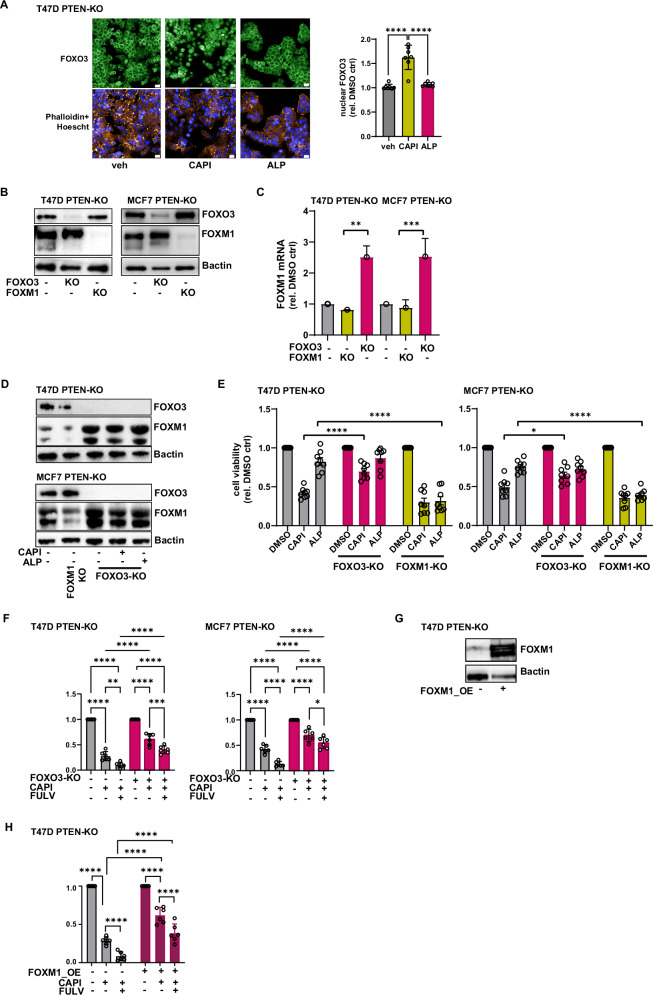
Sustained FOXM1 expression following FOXO3 depletion confers resistance to AKT inhibition. **A** FOXO3 immunofluorescence staining and quantification of its localisation in T47D PTEN-KO cells treated for 3 days with DMSO, 0.5 μM capivasertib and 0.5 μM alpelisib. Images were captured using the confocal microscope Yokogawa CV8000, 63× magnification. Immunofluorescences were performed to visualise FOXO3 localisation using a specific secondary antibody (AF488) (green) and F-actin with DyLight 594 Phalloidin (orange). Nuclei were stained with Hoechst (blue). Scale bar 20 µm. Data was normalised to vehicle and plotted as mean ± SD. Statistical analysis one way ANOVA vs vehicle-treated, *p ≤ 0.05, **p ≤ 0.01, ***p ≤ 0.001, ****p ≤ 0.0001. **B** Characterisation by western blot of T47D PTEN-KO and MCF7 PTEN-KO where FOXM1 and FOXO3 were depleted after 3 days from guides transfection. β actin was used as loading control. **C** FOXM1 mRNA analysis in PTEN-KO MCF7 and T47D cells following FOXO3 or FOXM1 depletion (after 3 days from transfection). Data normalised to CTRL. Data are presented as mean of *n* = 2 ± SD. Statistical analysis *t* test, *p < 0.05, **p ≤ 0.01, ***p ≤ 0.001, ****p ≤ 0.0001. **D** Characterisation by western blot of T47D PTEN-KO FOXO3-KO and MCF7 PTEN-KO FOXO3-KO cells treated 96 h with DMSO, capivasertib and alpelisib (MCF7 PTEN-KO/FOXO3-KO treated with 1 μM capivasertib and 1 μM alpelisib; T47D PTEN-KO/FOXO3-KO treated with 0.5 μM capivasertib and 0.5 μM alpelisib). Lysates from PTEN-KO CTRL and FOXM1-KO cells were used as control. βactin was used as loading control. **E** Cell viability assay of T47D PTEN*-*KO/ FOXO3-KO and T47D PTEN-KO/ FOXM1-KO and MCF7 PTEN-KO/ FOXO3-KO and MCF7 PTEN-KO/ FOXM1-KO after 5-day treatment with DMSO, 1 μM capivasertib and 1 μM alpelisib (MCF7); and 0.5 μM capivasertib and 0.5 μM alpelisib (T47D). Data was normalised to DMSO; plotted as mean ± SD (*n* = 3). Statistical analysis 2-way ANOVA test vs vehicle-treated, *p ≤ 0.05, **p ≤ 0.01, ***p ≤ 0.001, ****p ≤ 0.0001. **F** Cell viability assay of T47D PTEN-KO/ FOXO3-KO / Ctrl KO after 5-day treatment with DMSO, 0.5 μM capivasertib and 100 μM fulvestrant. Data was normalised to DMSO treatment; statistical analysis one-ways ANOVA test vs vehicle-treated plotted as mean ± SD *p ≤ 0.05, **p ≤ 0.01, ***p ≤ 0.001, ****p ≤ 0.0001 (*n* = 3). **G** Characterisation by western blot of FOXM1 protein levels, lysates from *PTEN-*KO cells modulated to transiently overexpress FOXM1 (FOXM1_OE) or GFP (GFP_OE). βactin was used as loading control. **H** Cell proliferation assay (Day 5) of T47D PTEN-KO FOXM1_OE or GFP_OE after treatment with DMSO, 0.5 μM capivasertib monotherapy and 0.5 μM capivasertib + 100 nM fulvestrant (combination). Data was normalised to DMSO; plotted as mean ± SD *p ≤ 0.05, **p ≤ 0.01, ***p ≤ 0.001, ****p ≤ 0.0001 (*n* = 6).

To gain insight into drivers of response to AKTi in ER+ BC cells downstream effectors were assessed in *PIK3CA*mut parental and PTEN-KO cell lines. One key effector regulated by PI3K-AKT signalling is FOXO3, where transcriptional activity is controlled by direct phosphorylation by AKT resulting in cytoplasmic accumulation and inhibition of its activity^29^. Following monotherapy treatment of T47D *PIK3CA*mut PTEN-KO cells with capivasertib for 3 days, FOXO3 translocated to the nucleus (Fig. 5A), whereas it was mainly cytoplasmatic after monotherapy treatment with the PI3Kαi alpelisib (Fig. 5A).

The relative role of FOXM1 and FOXO3 in mediating sensitivity to PI3Kαi or AKTi in *PIK3CA*mut cells with compromised PTEN levels was assessed by reducing expression of FOXM1 or FOXO3 by transfecting sgRNA guides (CRISPR/Cas9) (Fig. 5B). Growth of *PIK3CA*mut PTEN-KO T47D and MCF7 cells when *FOXO3* (FOXO3-KO) or *FOXM1* (FOXM1-KO) were depleted was similar to control cells, although FOXO3-KO resulted in a small statistically significant growth advantage compared to FOXM1-KO (Supplementary Fig. 3D).

In *PIK3CA*mut PTEN-KO cells *FOXO3* depletion resulted in increased FOXM1 mRNA (Fig. 5C), as previously described^30^. In *PIK3CA*mut PTEN-KO cells *FOXO3* depletion followed by capivasertib treatment for 5 days lead to sustained FOXM1 protein expression, with FOXM1 protein levels similar to alpelisib or DMSO treatments, confirming that FOXO3 regulates FOXM1 expression (Fig. 5D). The sustained FOXM1 expression in *FOXO3* depleted cells reduced capivasertib anti-proliferative activity in *PIK3CA*mut PTEN-KO cells compared to *PIK3CA*mut PTEN-KO cells expressing FOXO3 (Fig. 5E). Again, this was similar to alpelisib or vehicle treatment (Fig. 5E). Finally, in PTEN-KO/Ctrl cells expressing FOXO3 the combination of fulvestrant and capivasertib versus capivasertib monotherapy resulted in a substantial impact on cell viability with a 60–70% reduction (Fig. 5F), this decrease was diminished to a 20-30% reduction in PTEN-KO/FOXO3-KO cells (Fig. 5F).

We next assessed if modulation of FOXM1 expression itself could impact capivasertib efficacy (Fig. 5E). Reducing FOXM1 expression did not further enhance the effect of capivasertib in *PIK3CA*mut PTEN-KO cells (Fig. 5E). However, *FOXM1* depletion enhanced sensitivity to PI3Kα (alpelisib) in the PI3Kαi insensitive *PIK3CAmut* PTEN-KO control cells (Fig. 5E) resulting in growth inhibition similar to AKT inhibition (capivasertib), confirming the role of FOXM1 in attenuating sensitivity to PI3Kαi’s^14^. Next FOXM1 or control GFP was overexpressed in T47D PTEN-KO cell lines (FOXM1_OE, GFP_OE) (Fig. 5G). Overexpressing FOXM1 reduced capivasertib anti-proliferative effects in T47D PTEN-KO cells when compared to control cells expressing GFP (Fig. 5H). Results are similar to those previously described for PI3Kα inhibition^14^.

### Chronic capivasertib exposure results in sustained FOXM1 expression in *PIK3CA-*mutant cells

**Fig. 6:**
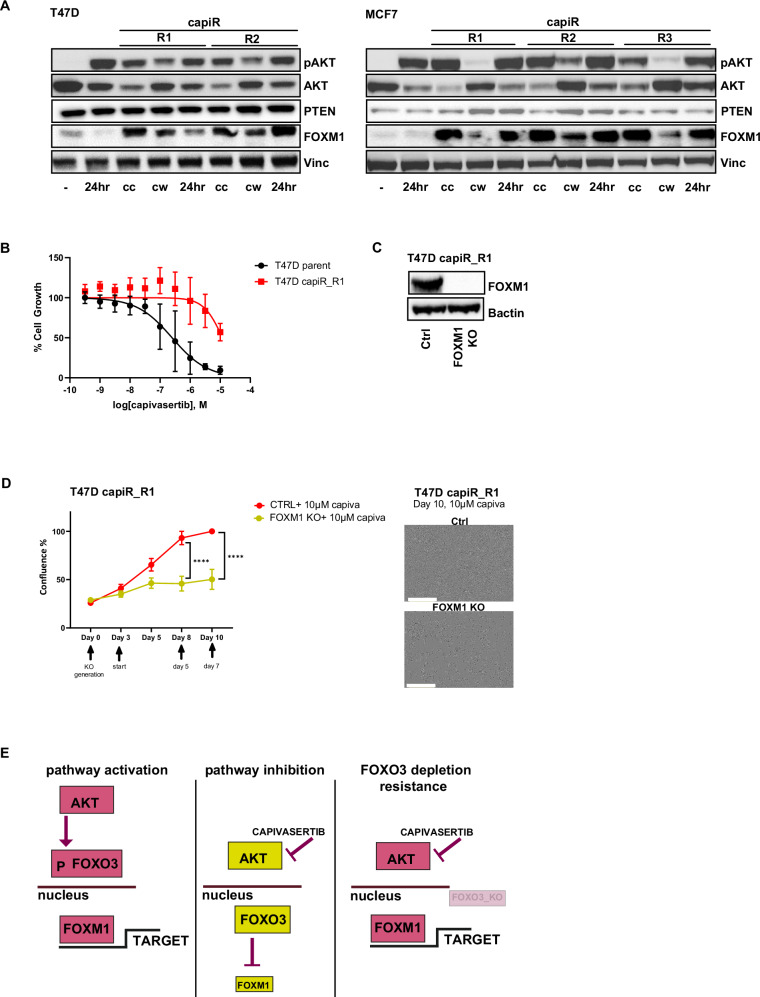
Increased FOXM1 protein expression in resistant capivasertib breast cancer cell lines. **A** Characterisation by western blot of T47D and MCF7 capivasertib resistant lines (capiR) compared to T47D and MCF7 parental cell lines. 2 or 3 pools were generated per each cell line (capiR R1, R2 or R3). Vinculin was used as loading control. Parental cells +10 μM capivasertib (24 h), capiR with continuous 10 μM capivasertib treatment (cc), capiR without capivasertib treatment for 120 h (cw), capiR without capivasertib treatment for 96 h and re-dosing (10 μM capivasertib) for 24 hours (24 h). **B** Dose response graphs of T47D capiR R1 line treated for 7 days with 10 μM capivasertib. Dose responses were calculated using Graphpad Prism. **C** Characterisation by western blot of FOXM1_KO vs control_KO in T47D capiR R1 line (expressing Cas9). βactin was used as loading control. **D** Depletion of *FOXM1* (FOXM1_KO) in T47D capiR R1 cells. 3day-KO followed by a treatment with 0.5 μM capiva growth curve plotted as mean ± SD (*n* = 3). Statistical analysis 2-way ANOVA test vs CTRL *p ≤ 0.05, **p ≤ 0.01, ***p ≤ 0.001, ****p ≤ 0.0001. Representative images plotted for each time point and condition. Scale bar 400 µm. **E** Schematic graph showing AKT, FOXM1 and FOXO3 roles in PI3K/AKT pathway inhibition.

To explore the consequences of long-term exposure to capivasertib a panel of capivasertib tolerant or resistant T47D and MCF7 cell pools were generated by continuous capivasertib treatment at 10μM for up to 6 months (capiR) (Fig. 6A). In the two T47D cell pools (capiR R1, R2) and three MCF7 cell pools (capiR R1, R2 and R3) growing under continuous 10μM capivasertib exposure resulted in an increase in FOXM1 protein expression relative to the parental lines (Fig. 6A). FOXM1 expression remained higher versus parental lines both upon capivasertib withdrawal for 96 h, re-treatment for 24 h, continuous dosing (cc) or withdrawal for 120 h (cw). To confirm that sustained FOXM1 expression influences the response to capivasertib in capiR cells, we transfected FOXM1 target sgRNA guides in the T47D *PIK3CA*mut Cas9 capiR R1 pool (Figs. 6B, 6C). A decrease in FOXM1 expression in capiR R1 cells led to reduced cell proliferation under 10 μM capivasertib treatment, compared to the growth of the resistant capiR R1 cells (Fig. 6D).

Overall, these data demonstrate that in ER+ BC with alterations in *PIK3CA*, AKT directly controls the FOXO3-FOXM1 axis. Capivasertib FOXO3 deregulation results in the release of the repression of the direct transcriptional target FOXM1, which contributes to the anti-proliferative effects of capivasertib alone and in combination with fulvestrant (Fig. 6E). Moreover, AKT inhibition is not effective if FOXO3 expression is compromised as it prevents release of the repression of FOXM1. These findings also highlight the importance of FOXO3 and FOXM1 as biomarkers for prediction of drug resistance or sensitivity respectively when targeting the PI3K-AKT pathway in ER+ BC (Fig. 6E).

## Discussion

ER+ BC tumours with alterations in *PIK3CA* are sensitive to PI3Kαi and AKTi, while those with alterations in *PIK3CA* and *PTEN* are sensitive to AKTi, with enhanced therapeutic benefit when they are combined with inhibitors of ER signalling^7,8^. While PI3Kα is activated by mutations or amplifications in *PIK3CA* that increase or sustain activation, there are multiple mechanisms that influence PTEN function such as genetic loss, alterations or epigenetic events that lead to down-regulation or inactivation of PTEN protein. All these alterations can modify how PI3K signalling is regulated, and tumour cells can have multiple alterations that influence PI3K-AKT signalling.^18,31^. Here we show that a subset of ER+ *PIK3CA* altered BC have numerous homozygous and hemizygous alterations in *PTEN* that can lead to attenuation of PTEN function. In pre-clinical ER+ *PIK3CA* models we found that homozygous and hemizygous alterations that ablate or reduce PTEN protein can attenuate response to PI3Kαi but not to AKTi alone and in combination with fulvestrant. These data indicate that in ER+ BC there appears to be a linear link between PI3K and AKT, with sensitivity to alpelisib and capivasertib both dependent on the ability of each compound to drive modulation of FOXO3 transcription and secondary regulation of FOXM1. Moreover, loss of FOXO3 results in resistance to capivasertib reducing anti-proliferative effects, which are reversed up on deletion of FOXM1. This suggests that FOXO3 is a pivotal effector of both PI3Kαi and AKTi in *PIK3CA* altered breast cancer.

PTEN protein plays a fundamental role in PI3K-AKT pathway regulation^32^ and inactivating *PTEN* via mutation or deletion is one of the most common somatic events in human cancer^33^. *PTEN* is frequently lost, entirely affecting both copies of the gene (homozygous loss) or by affecting one copy of the gene (hemizygous loss)^22^. It had been suggested that mutations in *PIK3CA* and *PTEN* tend to present in a nearly mutually exclusive pattern in breast tumours^21^. However, our analysis based on publicly available datasets METABRIC, TCGA and TEMPUS showed that 3–9% of the samples classified as ER+/HER2- BC harbour concurrent alterations in both *PIK3CA* and *PTEN* (homozygous loss, missense mutations or truncations), in line with a series of data reporting concomitant alterations of the two genes in breast cancer^34,35^, and in the recent Phase III CAPItello 291 clinical trial^8^.

PTEN is a haplo-insufficient tumour suppressor with partial loss of PTEN function sufficient to modulate pathway activation and promote tumour development [16]. Therefore, the observation of broader hemizygous mutations that increase the overall frequency of *PTEN* gene alterations in ER+/HER2- BC biopsies with *PIK3CA* activating mutations across different clinical datasets is intriguing. Alterations in the *PTEN* gene are commonly identified by next-generation sequencing, with partial or entire *PTEN* homozygous gene deletion qualifying as an alteration. In all the clinical cohorts examined hemizygous *PTEN* deletions were much more frequent than homozygous deletions in ER+/HER2- BC biopsies with *PIK3CA* activating mutations, although the precise frequency rates for hemizygous *PTEN*del might be sightly overestimated as a result of the sequencing technique used in each study. Therfore it is clearly possible that both *PTEN* hemi- and homozygous alterations in *PTEN* may influence treatment response in tumours with *PIK3CA* activating mutations. Importantly our analysis of *PTEN* status in ER+/HER2- BC *PIK3CA* mutant biopsies did not include *PTEN* inversions, fusions, promoter methylations, and large-scale structural variants, which also impact protein levels. Taking this into account it is possible that PTEN protein function may be more variable across ER+ BC than previously thought. Further parallel analysis of patient cohort for homo- and hemizygous alterations in *PTEN*, secondary regulation of transcription, and relationship to PTEN protein levels and function in tumours will be important.

As expected, ER+ breast cancer preclinical models harbouring *PIK3CA* activating mutations^26,27,36,37^ were equally sensitive to PI3Kα (alpelisib) or AKT (capivasertib) inhibition. PTEN protein loss is an alternate mechanism of PI3K activation and has been correlated with progressive disease on PI3Kαi’s^18,31^. PTEN protein loss is also correlated with AKT activation through different PI3K isoforms^38^ therefore loss of PI3Kαi sensitivity in PTEN protein null models is not necessarily surprising^18,23–26^. However, it is intriguing that a small reduction in protein expression in the hemizygous models is also able to attenuate sensitivity to PI3Kαi similar to the homozygous loss. This suggests that smaller variations in PTEN than expected could be important in the context of other driver mutations such as *PIK3CA*. It was also interesting that despite some increase in basal AKT signalling activation in the double mutant cell line models there was not an increase in the dependency on AKT signalling.

In addition to loss of PTEN protein other secondary mutations in the p110α catalytic pocket, Q859K, Q859H, and W780R, can provide resistance to PI3Kαi’s (alpelisib and inavolisib/GDC-0077)^39^. This collectively suggests that reactivation of AKT signalling is critical for these tumour cells, indeed treatment with the allosteric AKTi miransertib (ARQ092) and the ATP-competitive AKTi ipatasertib re-sensitised tumour cells expressing these double mutants^39^. Hence, combining an ER antagonist with AKTi’s may not be liable to the same resistance mechanisms as PI3Kαi’s, and can prevent the reactivation of oncogenic signalling through the PI3K-AKT pathway to deliver broader therapeutic potential.

The analysis of different cell lines implicated the transcription factors FOXO3 and FOXM1 as mediators of the efficacy of capivasertib and alpelisib in *PIK3CA* mutant ER+ BC cell lines. The forkhead transcription factor FOXM1 is a direct transcriptional target repressed by the forkhead protein FOXO3, a critical downstream effector of the PI3K-AKT-FOXO signalling pathway^40^. AKT mediated phosphorylation of FOXO3 results in its cytoplasmic accumulation, and consequent release of the repression of the potent oncogene FOXM1^29^. PI3K, AKT, and dual PI3K/mTOR inhibitors enhance FOXO3 nuclear localisation in breast cancer cells which is implicated in both sensitivity and feedback mediated resistance to pathway inhibition^14,15^. In PI3Kα resistant-*PIK3CA*mut/PTEN-KO cells combined PI3Kα and PI3Kβ blockade resulted in pathway inhibition, FOXO3 relocation to the nucleus and inhibition of FOXM1 expression^14^. Consistent with this being AKT dependent, in the PI3Kα resistant-*PIK3CA*mut/*PTEN*-KO cells inhibition of AKT level drives FOXO3 nuclear relocation, which in turn down regulates FOXM1 and its target genes involved in cell cycle progression^28^. FOXO translocation to the nucleus can drive upregulation of proteins such as IGF1 and RICTOR^41–43^ that can also drive PI3K-AKT pathway reactivation. Loss of FOXO3 expression drives resistance to capivasertib both alone and in combination with fulvestrant. One other study has associated FOXO3 downregulation as a resistance mechanism to PI3Kαi’s in ER+ BC^44^. Therefore, FOXO has biphasic effects, first acute translocation driving efficacy, and then second transcriptional effects that become evident upon constant pathway suppression that may attenuate the initial impact on growth.

FOXO translocation can be influenced by other mechanisms including other kinases. For example, the AGC kinase SGK1, also phosphorylates FOXO preventing nuclear translocation, and is increased in PI3Kαi resistant cell lines and tumours from patients refractory to PI3Kα inhibition^45^, as well as elevated expression being associated with reduced sensitivity to AKT inhibition in breast cancer cell lines^46^. When SGK1 expressing cells are treated with PI3Kα and PDK1 inhibitors, both AKT and SGK1 are inhibited, inducing tumour regression as a result of FOXO3 activation and mTORC1 inhibition^45^. Combinatorial treatment with endocrine therapy in this context also results in strong nuclear localisation of FOXO3^45^, which would inactivate FOXM1^47^. Therefore, tracking nuclear translocation of FOXO3 and down-regulation of FOXM1 expression may provide specific refined biomarkers for monitoring disease modification in *PIK3CA* altered ER+ BC.

We have used different approaches to confirm the importance of FOXM1 as part of mechanism of response to PI3K-AKT pathway inhibition in *PIK3CA*mut ER+ BC. Overexpression of FOXM1 in *PIK3CA*mut/PTEN-KO cells attenuated the anti-proliferative effects of capivasertib. Furthermore, *PIK3CA*mut cell pools rendered capivasertib resistant or tolerant following prolonged treatment (up to 6 months) exhibit increased FOXM1 protein expression, with downregulation of FOXM1 expression in the presence of capivasertib reducing proliferation of resistant cells. Collectively these show that FOXM1 regulation is important for the capivasertib response, and that lack of FOXM1 downregulation may attenuate response. Interestingly overexpression of FOXM1 did not completely ablate sensitivity to capivasertib in the cell pool. While this may reflect heterogeneity of expression within the population of cells following transfection, it is also possible the interplay between FOXM1 and FOXO is more complex with different FOXM1 isoforms playing subtly different roles, or other signalling pathway also being important. However following FOXM1 expression as a biomarker to gain insights into dependence on, and resistance to PI3K/AKT pathway inhibition in ER+ *PIK3CA* mutant breast cancer is important. FOXM1 may also be a therapeutic target in tumour progressing following PI3Kαi or AKTi treatment. It would be a clear effector when loss of FOXO3 expression/function is driving resistance, but also when other mechanisms are preventing translocation of FOXO to the nucleus, or attenuating the effects of PI3K-Akt pathway inhibitors. FOXM1 expression was not only modulated by FOXO3 translocation. Treating cells with fulvestrant in combination with AKTi resulted in increased reduction of FOXM1, exploring other mechanisms that may contribute to regulating FOXM1 levels will be important. The FOXO3-FOXM1 interplay aligned to PI3K-AKTi sensitivity will may be important to consider. It is possible that adaption driven by long term continuous pathway suppression could be mitigated by intermittent dosing strategies with PI3Kαi or AKTi, or treatment breaks.

In summary this study establishes a direct relationship between PI3Kα and AKT in *PIK3CA* altered ER+ BC where signalling converges on the AKT dependent FOXO3-FOXM1 axis as a pivotal response to pathway inhibition. In addition, the data suggest that further consideration of the PTEN protein status of *PIK3CA* altered ER+ BC tumours is warranted to understand how this modulates response to treatment. Modulation of FOXO3 is a key effector as treatment response is attenuated when FOXO3 does not reduce FOXM1 expression, resulting in sustained FOXM1 protein mediated cell cycle progression. Thus, modulation of FOXM1 levels may be an important factor in predicting response and resistance to PI3K-AKT inhibition in ER+ BC. The data suggests that targeting AKT is an effective strategy in ER+ BC preclinical models with *PIK3CA* mutations with or without compromised PTEN protein expression due to in part regulation of FOXO3 translocation driving PI3K-AKTi mediated cell cycle arrest in part through FOXM1 inhibition. These results add to our understanding of drivers of response to PI3K-AKT inhibition in ER+ BC and the value of FOXO3 and FOXM1 as pharmacodynamic biomarkers for drugs targeting the PI3K-AKT pathway alone or in combination.

## Methods

### Cell culture and reagents

Cell lines were authenticated using short tandem repeat (STR) profiling. Cells were cultured in a humidified incubator with 5% CO2 at 37 °C. MCF7 cells (*PIK3CA*_E545K) were cultivated with DMEM supplemented with 1% Glutamax and 10% FBS and T47D cells (*PIK3CA*_H1047R) with RPMI supplemented with 1% Glutamax and 10% FBS. Organoids derived from patient-derived xenografts were grown in DMEM/F12 (1:1 ratio) media supplemented with B27 (GIBCO), basic EGF (20 ng ml-1, Sigma), FGF (10 ng ml-1, Sigma), Heparin (4 μg ml-1, Sigma) with penicillin-streptomycin (1%) and normicin (1%).

All compounds were synthesised by AZ, capivasertib was synthesised according to^48^, and alpelisib was also purchased from Selleckchem. Compounds were dissolved in DMSO at a concentration of 10 mmol/L and administered at the following concentrations: capivasertib 1 μM in MCF7 cells and organoids and 500 nM in T47D cells; alpelisib 1 μM in MCF7 cells and organoids and 500 nM in T47D cells and fulvestrant was administered at 100 nM.

### Generation and validation of Cas9-expressing cell lines

MCF7, T47D and T47D capiR R1 cells were transduced with a lentivirus produced from the pKLV2-EF1a-Cas9Bsd-W vector [34]. At 72 h after transduction, cells were selected with blasticidin 2 μg/ml (ThermoFisher) and then single sorted into 96-well plates using serial dilution. Clonally derived lines were further expanded and analysed for Cas9 cutting activity using a Cas9 reporter assay, described previously in Dunn et al. 2022^49^. Briefly, cells were transduced separately with lentivirus produced with the following lentiviruses: pKLV2-U6gRNA5(Empty)-PGKBFPGFP-W and pKLV2-U6gRNA5(GFP gRNA)-PGKBFPGFP-W^49^. At 72 h after transduction, the ratio of BFP and GFP-BFP double-positive cells was analysed using flow cytometry using a LSR Fortessa instrument (BD) and FlowJo software. Cas9 activity in the cells (%) was calculated as (BFP-single positive cells)/(total number of BFP+ cells). All Cas9-cell lines used in this study had genome-editing Cas9-activity >90%.

### Generation of PTEN-KO, FOXM1-KO and FOXO3-KO cells

Cells expressing Cas9 were used to generate for the *PTEN, FOXM1*, and *FOXO3* KO cells. PTEN gRNAs were designed using Yusa Human CRISPR library V1 (Addgene #67989) and cloned into the lentiviral plasmid pKLV-U6gRNA (BbsI)-PGKpuro2ABFP (Addgene #50946). Three days after transduction, puromycin (2 μg/ml) (Sigma–Aldrich) was added to the media for 7 days to kill non-transduced cells. Control cells were infected with the pLentiV2 lacking the guide.

FOXM1 and FOXO3 gRNA were obtained from horizondiscovery.com. gRNAs were transfected into cells using Lipofectamine RNAiMAX (1%). The serum-free transfection medium contained RPMI media (phenol red free) + 1% Glutamax. The medium was changed after 3 days. The guides RNAs are listed in Methods Table 1.

**Table 1:** Oligonucleotides

GENE	SEQUENCE
PTEN gRNA 1	5′ ACTTTGATATCACCACACAC 3′
PTEN gRNA 2	5′ AGAGCGTGCAGATAATGACA 3′
FOXM1 gRNA 1	5′ ATGGGCAGCGTTTCCTTAAT 3′
FOXM1 gRNA 2	5′ GGCAATGGCACCTTCACCGA 3′
FOXM1 gRNA 3	5′ TATCATGGCCATGTAAGAGT 3′
FOXM1 gRNA 4	5′ TTGGCAATGTGCTTAAAGTA 3′
FOXO3 gRNA1	5′ ACTGCCACGGCTGACTGATA 3′
FOXO3 gRNA2	5′ TCGCCCTTATCCTTGAAGTA 3′
FOXO3 gRNA3	5′ ACAGAGTGAGCCGTTTGTCC 3′
FOXO3 gRNA4	5′ CCTGCCATATCAGTCAGCCG 3′
OR1A1 gRNA1	5′ GATGGTTGGCCAGCATCTTA 3′
OR1A1 gRNA2	5′ ATGCTGGCCAACCATCTCTT 3′
OR1A1 gRNA3	5′ GTTGGCCAGCATCTTAGGGA 3′
OR1A1 gRNA4	5′ GGTAATGTCACAGTAGAAGT 3′

### Generation of FOXM1_OE and GFP_OE T47D cells

T47D PTEN-KO cell lines were expanded in RPMI 1640 growth media (Thermo Fisher Scientific, cat: 11875093) supplemented with 10% Foetal Bovine Serum (Corning, cat: 35-015-CV) and 1% Glutamax (Thermo Fisher Scientific, cat: 35050061). A DNA fragment encoding either the human FoxM1 gene (UniProt: Q08050-1) or GFP (UniProt: P42212) was chemically synthesised by GeneArt and the sequence optimised for expression in Homo sapiens. These fragments were cloned into pcDNA 3.1(+) with EcoR1 and Xhol restriction sites by GeneArt (Thermo Fisher Scientific).

Electroporation was performed using the MaxCyte Stx system according to manufacturer’s guidelines. Cells were harvested at 65% confluency with 1 × TrypLE Select (Thermo Fisher Scientific, cat: 12563011) and the pellet washed twice in Electroporation Buffer (Cytiva cat: EPB5). Cells were resuspended to a density of 1 × 10^8^ cells/ mL and added to OC-400 RUO cuvettes (Maxcyte, cat: SOC-4) for electroporation using the ‘Renca’ parameters. Cells were recovered by seeding into a Nunc TC treated T75 flask (Thermofisher Scientific, cat: 156499) without media and place in an incubator (37 °C, 5% CO2) for 30 min. Cells were then resuspended in 5 mL media and counted prior to plating. Cells were seeded in 96-well plates (Sigma, Gillingham, Corning Costar #3904), 4000/ well in 100 µl and incubated at 37 °C, 5% CO2 in the Incucyte (Images were taken every 4 h) (Sartorius, UK). Cell confluence was plotted using GraphPad PRISM 8.

### Generation of capivasertib resistant cell lines (capiR)

To generate resistant cell populations parallel cultures of parental cell lines were exposed to escalating concentrations of capivasertib over 6 months up to a final dose of 10 μM which generated 2 T47D (capiR R1, R2) and 3 MCF7 capiR cell pools (capiR R1, R2, R3).

### Cell viability assay

Cells were seeded in 96-well plates (Sigma, Gillingham, Corning Costar #3904). MCF7 and T47D cell lines were seeded at 2000 cells/well in 90 µl and organoids at 20000 cells/well in 90 µl. After 24 h, they were drug-treated 120 h. Proliferation was measured using AlamarBlue (Invitrogen) diluted 1:10. After reagent addition (10 µl/well), cells were incubated for 4 h at 37 °C and organoids were incubated overnight at 37 °C. Fluorescence was measured using a Envision reader (Perkin Elmer) at 530 nm with 590 nm as a reference wavelength.

### Incucyte proliferation curves

Cells were seeded in 96-well plates (Sigma, Gillingham, Corning Costar #3904), 2000/ well in 100 µl and incubated at 37 °C, 5% CO_2_ for 8 days in the Incucyte (Images were taken every 4 h) (Sartorius, UK). Cell confluence was plotted using GraphPad PRISM 8.

### Flow cytometry

Click-iT EdU Alexa Fluor 647 Flow Cytometry Assay Kit (C10424; Life Technologies) was used for staining, according to the manufacturer’s protocol. Cells were plated at 1.5 × 105/well in 6 well plates and then treated with EdU for 1 h, trypsinized and filtered through 40-μm cell strainers to obtain single cells. DAPI (Thermo Fisher) was used at 1ug/mL. Cell sorting was performed on a FACSymphony (Becton Dickinson, USA). Cell cycle gating was carried out using FlowJo_v10.8.0 software, and cell cycle distributions were plotted using GraphPad PRISM 8.

### Immunofluorescence

Cultured T47D cells were seeded (1000 cells/90μL) in a 384-well plate and treated for 5 days. Cells were then washed in PBS, fixed in 4% PFA for 30 min at RT, washed three times in PBS, permeabilized with PBS + 0.1% Triton-X for 20 min, washed 3 × 10 min in PBS, and then incubated in 5% BSA in PBS for 1 h at room temperature. Cells were then incubated with specific primary antibodies at 4 °C (Methods Table 2) overnight, washed three times with PBS, and then incubated with secondary antibody AlexaFluor 488 goat anti-mouse IgG (1:500 dilution) (A32723 Invitrogen) and Hoechst (1:2000) for 1 h at room temperature. DyLight 594 Phalloidin (Cell signalling) was used to label F-actin (1:20) 15 min at RT. After three washes with PBS images were taken using a confocal microscope (Yokogawa CV8000).

**Table 2:** Antibodies

Antibody_Brand_ Cat. Number	DILUTION:
ERα _ThermoFisher SP1 #9101S	1:400
pAKT S473 CST #4060	1:1000
AKT CST #9272	1:1000
pPRAS40 CST #2997	1:1000
PRAS40 CST #2610	1:1000
pS6 CST #2211	1:1000
S6 CST #2217	1:1000
PTEN CST #9559	1:1000
Vinculin CST #13901	1:1000
βactin CST #4970	1:5000
FOXM1 CST #20459	1:500
FOXO3 CST #99199	1:1000 IF 1:200

### In vivo tumour xenograft and PDX models

All animal work was conducted according to AstraZeneca’s Global Bioethics Policy (https://www.astrazeneca.com/content/dam/az/Sustainability/Bioethics_Policy.pdf), in accordance with the PREPARE guidelines and reported in line with the ARRIVE guidelines.

PDX models characteristics: CTG3283 (*PI3KCA*_G1049R and *PTEN* hemizygous deletion)^23,25^ and ST3932 (*PIK3CA*_R88Q and *PTEN* homozygous deletion)^24,26^, T272 (*PIK3CA*_E39K)^27^ and CTC174 (*PIK3CA*_N345K)^36,37^. CTG3283 was licensed from Champions Oncology, and studies were performed internally at AstraZeneca (Boston, USA) in AAALAC-accredited facilities. Animal studies were performed in accordance with protocols approved by the IACUC, AstraZeneca R&D (Boston) in compliance with the Guide for the Care and Use of Laboratory Animals, 8^th^ Edition (National Research Council, National Academies Press, Washington, D.C., USA). Female NSG mice, aged 5–6 weeks, were purchased from The Jackson Laboratory and animals were housed at five animals per individually vented cage, enriched with corncob bedding, nesting material and solid plastic enrichment tubes. Animals were acclimatised for a week before entering studies. Animals were identified by LabStamp®. The housing room temperature was 22 ± 2 °C with humidity at 57.5 ± 17.5% with a 12 h light, 12 h dark cycle. 2 h light, 12 h dark cycle. Animals were fed ad libitum with an irradiated rodent diet and drinking water. Two days prior to tumour implant mice were given acidified water containing 0.007938 mg/mL 17β estradiol in amber bottles, which was continued throughout the study and refreshed every 5 days. Xenografts were established by mammary fat pad surgical implantation of ~30 mm^3^ tumour fragment into the right 2nd thoracic fat pad of 5- to 6-week-old female NSG mice under isoflurane anaesthesia. Tumours were allowed to reach 0.07–0.32 cm^3^ before being randomly assigned to study. CTG3283 study was run with n = 7 animals per arm. Tumour volume (mm^3^) was calculated as width² x length x 0.52. Studies with ST3932 were performed under contract with XenoStart (San Antonio, TX, U.S.A.) at AAALAC-accredited facilities and performed in accordance with protocols approved by the START ‘Institutional Animal Care and Use Committee’ (IACUC) and AstraZeneca’s ‘Platform for Animal Research Tracking aNd External Relationships’ (PARTNER) group. Female athymic Nude, outbred homozygous (Crl:NU(NCr)-Foxn1nu) mice aged 6–12 weeks were purchased from the Jackson Laboratory. Animals were housed at 4–6 animals per individually vented cages enriched with Corncob bedding, nesting sheets and plastic housing. Animals were identified by ear notch and or Lab Stamp® (Charles River). Animals were acclimatised for a minimum of 24 h before entering studies. The housing room temperature was 72 ± 2 °F with humidity-controlled at 45 ± 15% with a 12 h light, 12 h dark cycle. Animals were fed ad libitum with an irradiated rodent diet and drinking water. Drinking water was supplemented with β-oestradiol (8.5 mg/l) from when tumours were implanted. Xenografts were established by subcutaneous surgical implantation of ~70 mg tumour fragment into the right flanks of 6- to 12-week-old animals under anaesthesia (isoflurane). Tumours reached 0.15–0.3 cm^3^ before the animals were randomised into groups, *n* = 10 animals per arm except for fulvestrant and fulvestrant+capivasertib treatment *n* = 8. Tumour volume (mm^3^) was calculated as width² × length × 0.52. Studies with T272 were performed under contract with Xentech under authorisation by the ‘Direction Départementale de la Protection des Populations, Ministère de l’Agriculture et de l’Alimentation’, France and in accordance with protocols approved by Xentech along with AstraZeneca’s PARTNER group. Female athymic nude -Foxn1nu mice, aged 6–11 weeks, were purchased from ENVIGO, France. Animals were housed at three animals per c individually vented cage enriched with sterilised dust-free bedding cobs. Animals were identified via an RFID chip numbering system (Biolog Id TINY) and acclimatised for a week before entering studies. The housing room temperature was 24 ± 2 °C with humidity controlled at 55 ± 15% with a 14 h light, 10 h dark cycle. Animals were fed ad libitum with an irradiated rodent diet and drinking water. Drinking water was supplemented with β-estradiol (8.5 mg/l) from when tumours were implanted. Xenografts were established by subcutaneous surgical implantation of ~20 mm^3^ into the interscapular region under anaesthesia (Ketamine/Xylazine). Tumours reached 0.1–0.3 cm^3^ before animals were randomly assigned into treatment groups. Studies were run with *n* = 9 animals per arm. Tumour volume (mm^3^) was calculated as [length × width2]/2. CTC174 studies were performed at AstraZeneca in the United Kingdom under the authorisation of Home Office License PP3292652, reviewed by internal review teams. Female NSG mice aged 7–13 weeks were purchased from Charles River Labs UK and housed at five animals per individually vented cage, enriched with sterilised dust-free bedding, cardboard house, and wooden chew enrichment. Animals were acclimatised for a week before entering studies and identified via Ear Notch. The housing room temperature was 21 ± 2 °C with humidity controlled at 55 ± 15 °C with a 12 h light-cycle. Animals were fed ad libitum with an irradiated rodent diet and drinking water. Xenografts were established by surgical implantation of ~30 mm^3^ tumour fragment into mammary fat pad 9 under isoflurane anaesthesia. When tumours reached 0.25–0.3 cm^3^ animals were randomised into treatment groups, with *n* = 4 animals per arm. Tumour volume (mm^3^) was calculated as (Maximum measurement (length or width) × Minimum measurement (length or width) × Minimum measurement (length or width) × π)/6000.

Relative tumour volume (RTV) was calculated using the formula: RTV for day X = (Tumour volume on day X)/ (Tumour volume on day 0). Tumour growth inhibition (TGI) was calculated as follows: Percentage TGI on day X for treatment group = (((Vehicle RTV day X) − (Treatment group RTV day X))/ ((Vehicle RTV on day X) – (Vehicle RTV on day 0))) × 100.

Animals were randomised into treatment groups according to tumour size criteria outlined above to obtain treatment arms with homogeneous geomean volumes. Conscious animals were euthanised by cervical dislocation with secondary confirmation at the end of the study or for welfare condition.

Data are presented as treatment group geomeans, with error bars depicting SEM (calculated using GraphPad PRISM 8) as per AstraZeneca best practices.

### Formulations

Capivasertib was dissolved in DMSO (10% of final dosing volume), 1 M hydrochloric acid (2% of final dosing volume) and 25% kleptose (80% of final volume). The solution was adjusted to pH 5.0 (±0.1) then made up to final volume with 25% kleptose. Capivasertib was dosed twice daily (BID) 8 h apart by oral gavage 0.1 mL/10 g of the animal using a weekly schedule of 4 days dosing, 3 days not dosing at 100 mg/kg. Alpelisib was dissolved in 0.5% hydroxypropyl methylcellulose/0.1% Tween 80. Alpelisib was dosed once daily by oral gavage 0.1 mL/10 g of the animal dosing at 25 mg/kg. All solutions were replaced every 7 days and stored at room temperature in bottles protected from light. Fulvestrant was formulated once weekly as a suspension in peanut oil and dosed once weekly (QW) subcutaneously as a fixed dose of 0.1 mL/animal (5 mg/animal). Fulvestrant was dosed 1 h after the morning capivasertib or alpelisib oral dose when in combination.

### Patient-derived xenograft organoids (PDXOs)

Tissues from CTC174 and CTG3283 PDXs were treated with 0.1% collagenase I (ThermoFisher Scientific 17018029) for 4–6 h in Dulbecco′s Modified Eagle′s Medium (DMEM)/F12 (1:1 ratio, ThermoFisher Scientific 31330038) containing penicillin–streptomycin (1% ThermoFisher Scientific) and normocin (InvivoGen ant-nr-05) at 37 °C with gentle agitation. The reaction was stopped by adding 10% heat inactivated FBS (ThermoFisher Scientific A3840001), and the solution filtered using a 70 μm cell strainer. Cells were collected by centrifugation (500 rpm for 5 min) and grown in suspension in DMEM/F12 (1:1 ratio, ThermoFisher Scientific 31330038) culture medium supplemented with B27 (ThermoFisher Scientific, 17504044), basic epidermal growth factor (10 ng/mL, ThermoFisher Scientific PHG0266), fibroblast growth factor (10 ng/mL, ThermoFisher Scientific PHG0315), heparin (4 μg/mL, Sigma H3149-10KU) with penicillin–streptomycin (1%) and normocin (1%). The resulting organoids were used to perform cell viability assays. PDXOs were pelleted at 500 rpm for 5 min in 15 mL tubes. After discarding the supernatant, pellets were treated with TryPLE 10X (ThermoFisher Scientific A1217701) for 5 min, 3 mL culture media was added, and tubes centrifuged at 500 rpm for 5 min. 20,000 single cells were then seeded onto 96-well plates in culture media and analysed after drug treatment for 120 h. Cells were treated with 10% AlamarBlue (ThermoFisher DAL1025) and then incubated overnight at 37 °C. Two to three biological independent experiments were performed in three to four technical replicates. Fluorescence was excited at 530 nm and emission detected at 590 (PerkinElmer Multimode Plate Reader Envision). Drugs were used at the following concentrations: alpelisib (1 μM), and capivasertib (1 μM). Media containing the drugs was changed every 72 h.

### Western blot analysis

Proteins were extracted from cells using RIPA buffer (Thermo Fisher Scientific #89901), in the presence of protease (11836153001; Roche) and phosphatase (04906845001; Roche) inhibitors and 1:5000 Bezonase (Sigma, Gillingham, E1014). Protein concentration was determined in cleared lysates (BIORAD, Watford, 500–0112). Gel electrophoresis was performed using standard protocols with NuPAGE 4–12% Bis-Tris Midi Gels (Thermo Fisher Scientific WG1402BOX), run at 150 V for 90 mins. Transfers were carried out using the iBlot2 10-min 20 V, wet transfer overnight was performed to detect FOXM1 (40 V, ON). Membranes were cut into strips and probed with primary antibodies (Methods Table 2) diluted in TBS + 0.05% polysorbate (TBST) + 5% Marvel overnight, and then HRP-Goat anti-mouse secondary (CST #7076) or HRP-Goat anti-rabbit secondary (CST #7074) antibodies for 1 h. Antibody binding was detected using Pierce West Dura reagent and imaged using a Gbox (Syngene, Cambridge, UK). Band quantification was carried out in GeneTools (Syngene) and plotted in GraphPad PRISM 8. The identity, source, catalogue number, and dilution of the antibodies used in this study are summarised in Supplementary Methods.

### Gene expression analysis

Targeted gene expression analysis was performed using the BioMark HD Dynamic Array platform (192.24 dynamic array) and TaqMan assays (Invitrogen) following the manufacturer’s instructions Cells were washed with PBS and lysed in RLT buffer (Qiagen). RNA was extracted using the RNeasy 96 QIAcube HT Kit (Qiagen) with a DNAse digest as per the manufacturer’s recommendations. RNA was quantified using a Qubit Flex Fluorometer (Invitrogen), RNA purity was determined using a NanoDrop Eight (Thermo Scientific). All samples were diluted to 50 ng/µL. RNA was reverse transcribed and the resulting cDNA pre-amplified using TaqMan Assays (Thermo Fisher Scientific) for 14 cycles following Standard BioTools instructions. Preamplified material and Taqman Assays were loaded onto a 192.24 Dynamic Array IFC (Standard BioTools) as per manufactures guidelines and run on a Biomark HD (Standard BioTools). Ct values were analysed with Real-Time PCR Analysis software (Standard BioTools) and normalised to the average of selected housekeeping genes (dCt) (GAPDH, B2M, TFRC). Data was subsequently normalised to DMSO controls (ddCt). A two-sided Student *t* test was used to determine statistically significant changes, *p ≤ 0.05, **p ≤ 0.001, ***p ≤ 0.0001 and ****p ≤ 0.0001.

TaqMan Assays: Hs00179514_m1, Hs00983227_m1, Hs00938777_m1, Hs00187842_m1, Hs02786624_m1, Hs00951083_m1, Hs00765553_m1, Hs01030099_m1, Hs01084593_g1 (Thermo Fisher Scientific).

### Analysis of prevalence of *PIK3CA* and *PTEN* alteration in real-world datasets

*PIK3CA* and *PTEN* alteration prevalence was calculated in METABRIC (Molecular Taxonomy of Breast Cancer International Consortium, *n* = 2509)^5^, TCGA Firehose Legacy (The Cancer Genome Atlas, *n* = 1101) cBioPortal for Cancer Genomics and Tempus breast cancer datasets. METABRIC and TCGA data was taken from the public cBioPortal website (https://www.cbioportal.org/) and Tempus data was accessed via a strategic collaboration agreement (https://www.tempus.com/). Cohorts were restricted to ER+ /HER2- breast tumours which gave 459 sample for 437 patients in TCGA Firehose, 1382 samples and patients in METABRIC and 3327 samples in 3053 patients in Tempus. Hormone status was measured by immunohistochemistry for METABRIC and TCGA and generated by transcribing pathology reports for the TEMPUS cohort. *PTEN* driver mutations, homozygous deletions and heterozygous deletions were compared to *PIK3CA* driver mutations. Heterozygous loss calls for patients in the Tempus cohort were not readily available. Heterozygous loss calls are prone to false positives therefore the prevalence reported may be higher than the true value. Oncoprints and Venn diagrams were produced in R version 4.3.2 using ‘ComplexHeatmap’ and ‘Venneuler’ packages. CAPItello 291 data were extraced from^8^.

## Supplementary information

Supplementary file

Supplementary data

## Data Availability

All the other data supporting the findings of this study are available within the article and its Supplementary Information and Source data files and from the corresponding author upon reasonable request.
