# Supplementary material for: The interplay between FOXO3 and FOXM1 influences sensitivity to AKT inhibition in PIK3CA and PIK3CA/PTEN altered estrogen receptor positive breast cancer
Source: NPJ Breast Cancer. 2025 Apr 22;11:36. doi: 10.1038/s41523-025-00752-9 (PMC12015352; doi:10.1038/s41523-025-00752-9)
Supplement: Supplementary file 1 — Supplementary file [file 41523_2025_752_MOESM1_ESM.docx]

**Supplementary File**

**Supplementary figure 1.**

**
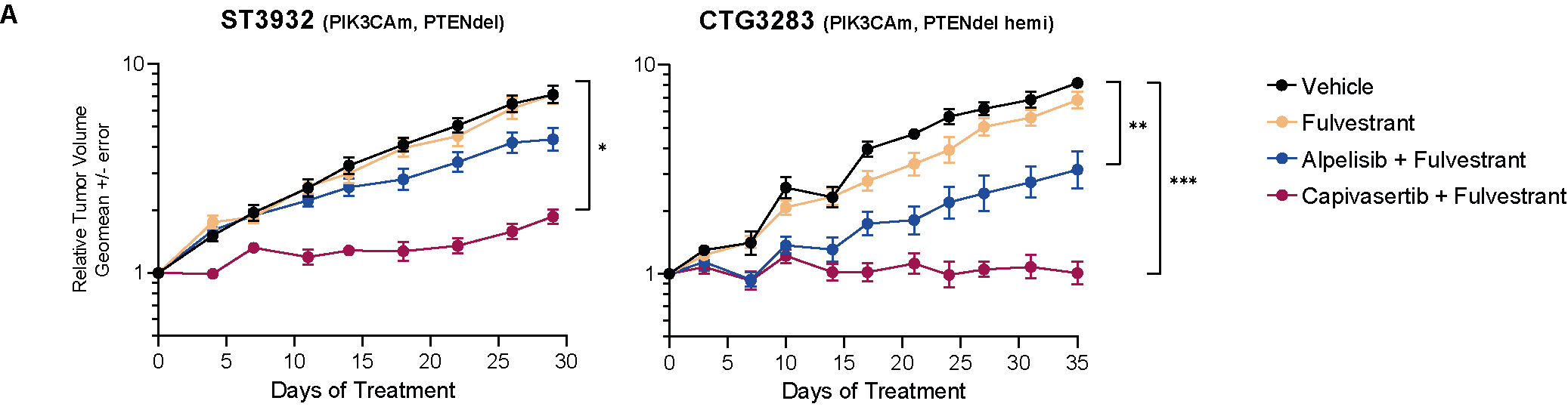
**

**(A)** In vivo activity of fulvestrant (orange circles) or combinations with alpelisib (dark red) or capivasertib (dark blue) in PIK3CAmut PTEN altered PDXs (CTG3283 n=7 and ST3932, n=8-10), control arms (closed circles) are shared with figure 3B. Treatments with 130 mg/kg capivasertib BID 4 days on 3 days off, 25mg/kg alpelisib QD, 5 mg/animal fulvestrant QW. Geomean tumour volumes ± SEM *p <= 0.05, **p <= 0.01, ***p <= 0.001, ****p <= 0.0001 are shown.

**Supplementary figure 2.**

**
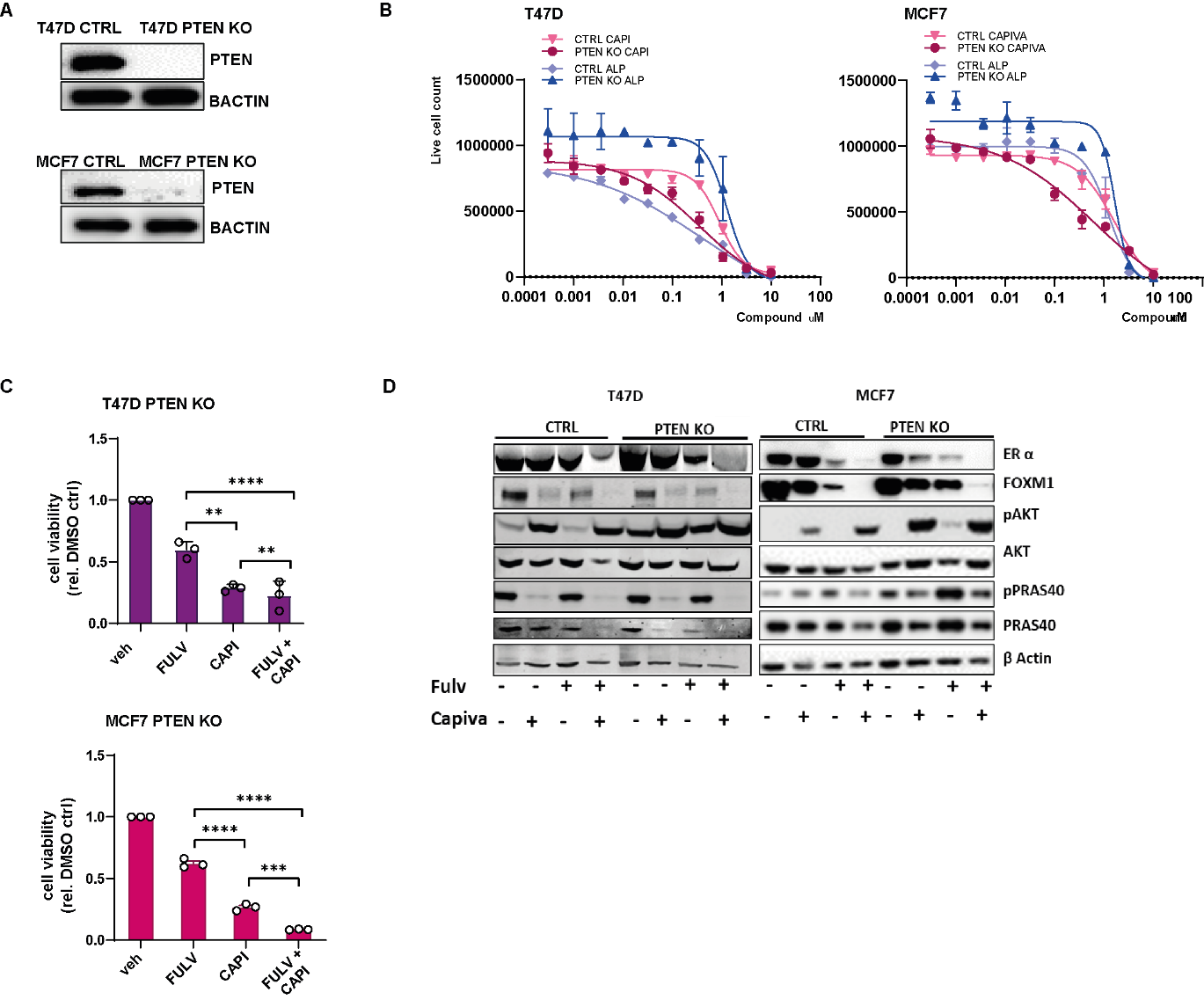
**

**(A)** Characterization of PTEN-KO cells used for the study by western blot. βactin was used as loading control. **(B)** Dose response graphs of all cells used in this study treated for 5 days with alpelisib and capivasertib. Dose responces were calculated using Graphpad Prism. **(C)** Cell viability assay of indicated T47D and MCF7cells after 5 days treatment with DMSO, fulvestrant, capivasertib and combination (CAPIVA+FULV). MCF7 PTEN-KO cells were treated with DMSO, 1μM capivasertib, 100nM fulvestrant, 1μM capivasertib + 100nM fulvestrant; T47D PTEN KO treated with DMSO, 0.5μM capivasertib, 100nM fulvestrant and 0.5μM capivasertib + 100nM fulvestrant. Data were normalised to DMSO; plotted as mean ± SEM (n = 3). Statistical analysis ANOVA test vs vehicle-treated, *p <= 0.05, **p <= 0.01, ***p <= 0.001, ****p <= 0.0001. **(D)** Characterization by western blot of MCF7 CTRL, MCF7 PTEN-KO, T47D CTRL and T47D PTEN-KO lysates after 96 hours treatment. MCF7 treated with 1μM capivasertib, 100nM fulvestrant, 1μM capiva+100nM fulv; T47D CTRL and T47D PTEN-KO treated with DMSO, 0.5μM capivasertib, 100nM fulvestrant and 0.5μM capiva + 100nM fulv. βactin was used as loading control.

**Supplementary figure 3.**


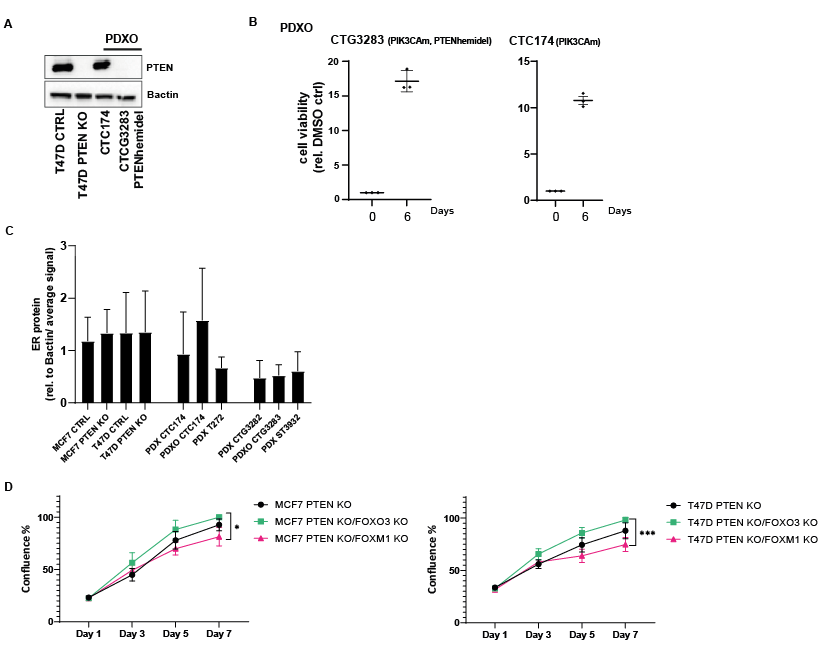


**(A)** Characterization by western blot of lysates from the PDXO models CTC174 and CTG3283. T47D CTRL and T47D PTEN-KO cell lysates were used as a control. β actin was used as loading control. **(B)** Cell viability assay shows cell growth between day 0 and day 6 in PDXO models. Data normalised to DMSO; data are plotted as mean ± SEM (n = 3). **(C)** Characterization by western blot of lysates from T47D CTRL, T47D PTEN-KO, MCF7 CTRL and MCF7 PTEN-KO, PDX and PDXO models CTC174 and CTG3283, PDX T272 and PDX ST3932. Averaged signal was used as a control. β actin was used as loading control. **(D)** Growth curves of MCF7 PTEN-KO, T47D PTEN-KO with/out double KO FOXM1 or FOXO3. Statistical analysis one way ANOVA vs CTRL+capi, *p <= 0.05, **p <= 0.01, ***p <= 0.001, ****p <= 0.0001.

**Supplementary Table 1.** Tables represent the proportion of different *PIK3CA* mutations in the *PTEN*alt (homozygous loss, missense mutations, truncations) ER+/HER2- breast tumours in TCGA (n=33) and Metabric (n=14) and PTEN HETLOSS (heterozygous loss) in TCGA (n=47) and Metabric (n=14). This is the extended version of Figure 1C.

**ER+HER2- BC *PIK3CA*mut *PTEN*alt**

| **TCGA** |  | **Metabric** |  |
| --- | --- | --- | --- |
| **mutation** | **%** | **mutation** | **%** |
| H1047R | 35.7 | H1047R | 33.3 |
| E542K | 14.3 | E545K | 21.2 |
| E545K | 14.3 | E542K | 9.09 |
| Q546R | 14.3 | E545K, E726K | 6.06 |
| E81K | 7.14 | H1047L | 6.06 |
| G1049R | 7.14 | E365K | 3.03 |
| Y1021C | 7.14 | E453, G460delinsD | 3.03 |
|  |  | E453, L455del | 3.03 |
|  |  | K111E | 3.03 |
|  |  | M1043V | 3.03 |
|  |  | N345K | 3.03 |
|  |  | Q546R | 3.03 |
|  |  | V105del | 3.03 |

**ER+HER2- BC *PIK3CA*mut *PTEN*del hemi**

| **TCGA** |  | **Metabric** |  |
| --- | --- | --- | --- |
| **mutation** | **%** | **mutation** | **%** |
| H1047R | 38.6 | H1047R |  |
| E545K | 22.8 | E545K | 37.6 |
| E542K | 10.5 | E542K | 18.3 |
| N345K | 3.51 | N345K | 9.68 |
| E110del | 1.75 | C420R | 9.68 |
| E542K,C901F | 1.75 | E103 | 4.3 |
| E542K,E726K | 1.75 | E110del | 1.08 |
| E970K | 1.75 | E453K | 1.08 |
| G1007R | 1.75 | E453K, Q546R | 1.08 |
| G118D | 1.75 | E545G | 1.08 |
| H1047L | 1.75 | E545K | 1.08 |
| H1047R,E365V | 1.75 | E726K | 1.08 |
| H1047R,P366R | 1.75 | G1049R | 1.08 |
| H1047y | 1.75 | H1047R, D1029H | 1.08 |
| M1043I | 1.75 | H1047R, E365K | 1.08 |
| M1043V | 1.75 | H1047R, E542A | 1.08 |
| Q546P | 1.75 | H1047R, P471A | 1.08 |
| Q546R | 1.75 | H450_P458del, H1065Y | 1.08 |
|  |  | K111E | 1.08 |
|  |  | M1043I | 1.08 |
|  |  | M1043V | 1.08 |
|  |  | Q546K | 1.08 |
|  |  | Q546P | 1.08 |
|  |  | R88Q | 1.08 |

**Main Western Blot Data**

**Figure 3A**

**Figure 4B**

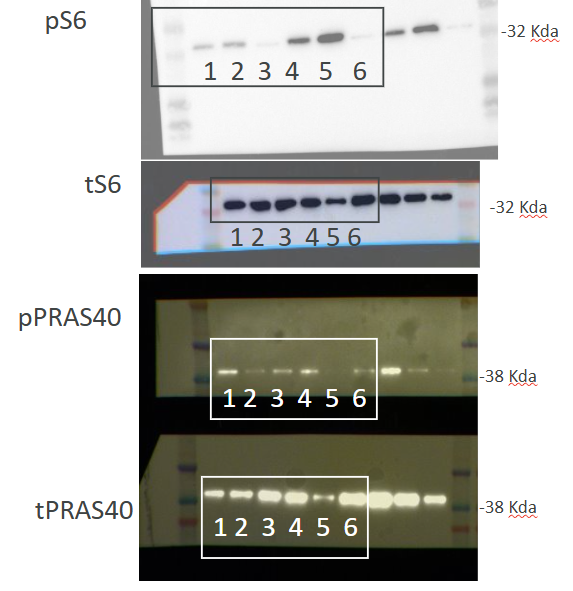

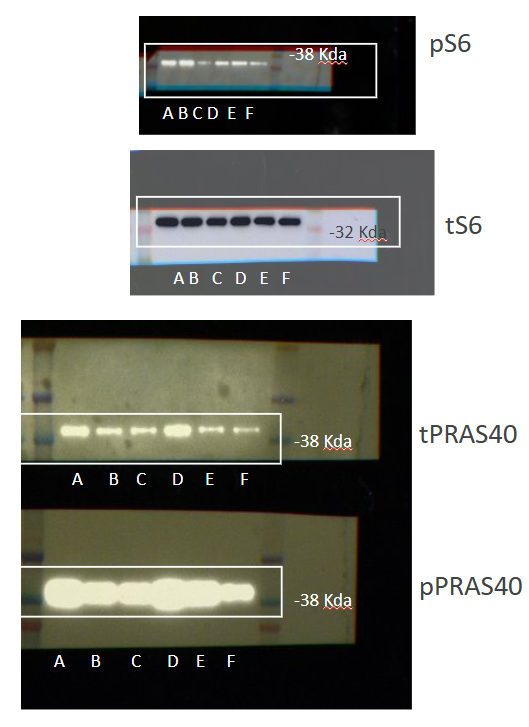


**Figure 4F**

**PDXO**


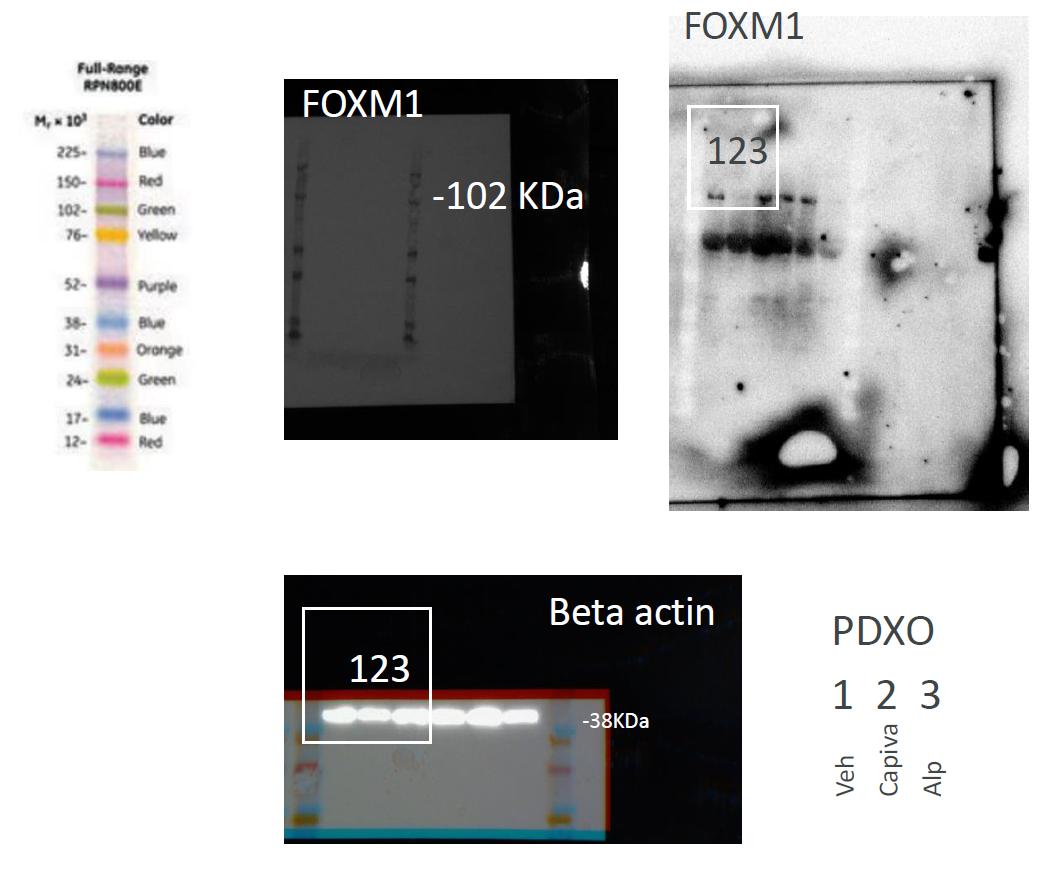

**
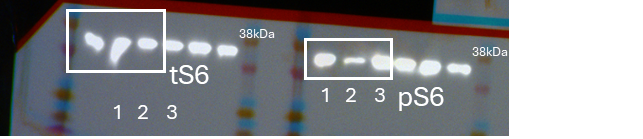
**

**Figure 4F**

**PDX**

**Fig 5B**

**Fig 5D**

**Fig 5G**

**Fig 6A**

**Vinculin**

**Fig 6C**

**Supplementary Western Blot data**

**Supl. Fig 2A**

**Supl. Fig 2D**

**Suppl. Fig. 3A**

Lane 4 = CTG3283

**Suppl. Fig. 3C**

ESR1 = ER protein
